# Profiling of the Tox21 10K compound library for agonists and antagonists of the estrogen receptor alpha signaling pathway

**DOI:** 10.1038/srep05664

**Published:** 2014-07-11

**Authors:** Ruili Huang, Srilatha Sakamuru, Matt T. Martin, David M. Reif, Richard S. Judson, Keith A. Houck, Warren Casey, Jui-Hua Hsieh, Keith R. Shockley, Patricia Ceger, Jennifer Fostel, Kristine L. Witt, Weida Tong, Daniel M. Rotroff, Tongan Zhao, Paul Shinn, Anton Simeonov, David J. Dix, Christopher P. Austin, Robert J. Kavlock, Raymond R. Tice, Menghang Xia

**Affiliations:** 1 NIH Chemical Genomics Center, National Center for Advancing Translational Sciences, National Institutes of Health, Rockville, MD 20850, USA; 2 National Center for Computational Toxicology, Office of Research and Development, U.S. Environmental Protection Agency, Research Triangle Park, NC 27711, USA; 3 Division of the National Toxicology Program, National Institute of Environmental Health Sciences, Research Triangle Park, NC 27709, USA; 4 ILS Inc., Research Triangle Park, NC 27709, USA; 5 Division of Bioinformatics and Biostatistics, National Center for Toxicological Research, US Food and Drug Administration, Jefferson, AR 72079, USA

## Abstract

The U.S. Tox21 program has screened a library of approximately 10,000 (10K) environmental chemicals and drugs in three independent runs for estrogen receptor alpha (ERα) agonist and antagonist activity using two types of ER reporter gene cell lines, one with an endogenous full length ERα (ER-luc; BG1 cell line) and the other with a transfected partial receptor consisting of the ligand binding domain (ER-bla; ERα β-lactamase cell line), in a quantitative high-throughput screening (qHTS) format. The ability of the two assays to correctly identify ERα agonists and antagonists was evaluated using a set of 39 reference compounds with known ERα activity. Although both assays demonstrated adequate (i.e. >80%) predictivity, the ER-luc assay was more sensitive and the ER-bla assay more specific. The qHTS assay results were compared with results from previously published ERα binding assay data and showed >80% consistency. Actives identified from both the ER-bla and ER-luc assays were analyzed for structure-activity relationships (SARs) revealing known and potentially novel ERα active structure classes. The results demonstrate the feasibility of qHTS to identify environmental chemicals with the potential to interact with the ERα signaling pathway and the two different assay formats improve the confidence in correctly identifying these chemicals.

A major public health concern is the potential disruption of normal endocrine function caused by the unwanted interactions of chemicals with steroid hormone receptors. Of particular concern are effects on estrogen receptors (ERs), which play a critical role in development, metabolic homeostasis, and reproduction^1^. In humans, there are two subtypes of ER, ERα and ERβ, which are encoded by distinct genes, ESR1 and ESR2, with different chromosomal locations^2^. Like other nuclear receptors, ERα and ERβ contain well-defined structural domains including a DNA-binding domain (DBD) and a ligand-binding domain (LBD)^3^. There are three primary endogenous ligands, estrone (E1), 17β-estradiol (E2), and estriol (E3). Among them, E2 is the predominant and most active estrogen in humans^4^ and binds to both ERα and ERβ ligand-binding domains with high affinity. Estrogenic effects occur through the numerous ER target genes that are either up- or down-regulated in response to ligand-induced activation of ERs.

Although ER signaling can be either ligand-dependent or ligand-independent^5^, many endocrine disrupting chemicals (EDCs) affect ER signaling by directly binding to the ER LBD. Such direct-acting EDCs include therapeutic agents, industrial chemicals, pesticides, and plasticizers^5^. For identifying ER agonists and antagonists, four types of *in vitro* assays are available: cell-free receptor binding assays and cell-based transactivation, translocation, or proliferation assays.

Cell-free receptor binding assays including radioligand-binding^6^ and fluorescence polarization^7^ are used to detect competition of chemicals with labeled ligands for receptors. These assays cannot distinguish agonists from antagonists or partial agonists from full agonists. To overcome these limitations, cell-based transactivation assays using reporter genes, such as β-lactamase (bla^8^) and luciferase (luc^9^), have been developed. These functional assays measure the ability of a chemical to induce or inhibit ER-dependent transcription through a reporter gene product. Two types of ER reporter gene cell lines are often used, one with a full-length ER (endogenous or recombinant transfected) in combination with a reporter gene and the other using a co-transfected receptor LBD/GAL4 DNA binding domain fusion protein and a reporter gene using the mammalian one-hybrid GAL4 system. To further study signaling events involved in ER activation, cell-based ER translocation assays have been developed using, for example, a green fluorescent protein chimera^10^. The MCF-7 cell proliferation assay has been widely used to study the mode of estrogen action *in vitro* and to detect weakly estrogenic compounds^11^. Among these assays, the cell-based reporter gene assays are commonly used in high-throughput screening^8^ due to their sensitivity, reproducibility, and ease of miniaturization.

As part of the Tox21 Phase II program^12^,^13^,^14^,^15^, we screened the Tox21 compound collection of ~10,500 chemicals (~8,300 unique) using two ERα reporter gene assays run in agonist and antagonist modes in a quantitative high-throughput screening (qHTS) format. One assay used the mammalian partial receptor one-hybrid system coupled to a β-lactamase reporter gene (ER-bla; HEK293 cell line) and the other assay used a full-length ER and luciferase reporter gene (ER-luc; BG1 cell line). The 10K compound library^16^ contains 88 compounds that are intentionally duplicated and sole-sourced to assess assay performance. Furthermore, the 10K library was tested in triplicate for each assay and the screening performance was evaluated by the reproducibility of the triplicate runs and the 88 duplicated compounds. The results from the ER-luc and ER-bla assays were compared and their ability to correctly identify ER agonists and antagonists was evaluated using a set of 39 reference compounds with known ER activity. The qHTS assay results were compared with results from ER binding assays^17^. Actives identified from both the ER-bla and ER-luc assays were analyzed for structure-activity relationships (SARs) revealing known and potentially novel ER-active structure classes.

## Results

### Assay performances and validation

**Figure 1:**
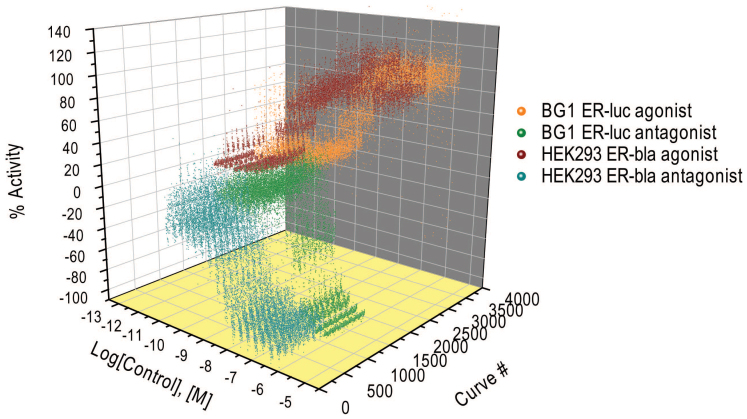
Concentration response data of the positive control compounds for the ER agonist (17β-estradiol) and antagonist (4-hydroxy tamoxifen) mode assays. The positive control compound is plated as 16-pt. titrations in duplicate in the control columns of every assay plate. In the figure, each concentration response curve is from one plate with a total of 153 plates per assay. The consistency of the control response curves is an indicator of good assay performance.

To identify chemicals that induce and/or inhibit ER activity, we screened the Tox21 10K library in both agonist and antagonist mode. Two cell-based assays, HEK293 ER-bla (LBD, partial receptor) and BG1 ER-luc (full length receptor) were used to screen the compounds at 15 concentrations. The antagonist mode assays were multiplexed with a cell viability readout to identify potential artifacts caused by cytotoxicity. Most assays performed well in the qHTS format with performance statistics^18^ including signal to background (S/B) ratios >3 fold, coefficient of variances (CVs) <10% and Z' factors >0.5, with the exception of the BG1 ER-luc agonist mode assay, which had a slightly lower S/B of 2.5 fold, and the HEK293 ER-bla antagonist mode assay, the Z' factor of which was 0.4 (Table 1). The positive control titrations embedded in every plate (17 β-estradiol for the agonist mode and 4-hydroxy tamoxifen for the antagonist mode assays) replicated well across the entire screen (Figure 1) with standard deviations (SDs) in AC50s <3 fold (Table 1).

As a measure of the assay performance, the reproducibility of 88 selected compounds (Tox21-88) plated as duplicates in the compound plates was evaluated^19^. The concentration at 50% activity (AC50) correlations (R^2^) of the duplicates that were active matches in the agonist mode screens were 0.83 for the HEK293 ER-bla assay and 0.80 for the BG1 ER-luc assay. The AC50 correlations (R^2^) of the duplicates that were active matches in the antagonist mode screens were lower for the HEK293 ER-bla assay (0.47) and higher for the BG1 ER-luc assay (0.76). To further evaluate assay performance, reproducibility was calculated for the HEK293 ER-bla, BG1 ER-luc, and the cell viability assays screened against the three copies of the 10K library with compounds plated in different well locations in each copy showing that these assays performed well with <1% mismatches in activity. The reproducibility measures of the three independent assay runs and the Tox21-88 in terms of active match, inactive match, inconclusive, and mismatch rates (see Methods section for details), and potency differences are listed in Table 2.

A set of 39 ER reference compounds (Suppl. Table 5) was used to further evaluate the reliability of these assays. These compounds are categorized as strong, moderate, or weak ER active or inactive. The number of ER reference compounds correctly and incorrectly identified by each assay was counted and the sensitivity and specificity of these assays were calculated based on these counts. The results are shown in Table 3. Both agonist mode assays performed well with >80% accuracies. It is interesting that the BG1 assay with a full length ER showed a higher sensitivity (96%) and lower specificity (67%) whereas the ER-bla assay with just the ER LBD showed a higher specificity (100%) and lower sensitivity (79%). These two assays were further compared in their ability to detect weak ER interacting compounds. The BG1 assay was able to identify 88% of the weak agonists, which is higher than the 71% identified by the ER-bla assay, consistent with the higher sensitivity shown by the BG1 assay (Table 3). The antagonist mode ER assays showed performances similar to the agonist mode assays, using only the six known antagonists to assess their performance, with an overall accuracy of 100% for the ER-bla assay and 91% for the BG1 assay. The difference between the accuracies of the two assays did not achieve statistical significance, which may have been a consequence of the small number of reference antagonist compounds available for evaluation. A few reference chemicals had inconclusive activity outcomes in the qHTS assays. These were reviewed manually and their final activity outcomes determined. The performance statistics recalculated including these inconclusive compounds are shown in Table 3 in parentheses. The overall accuracies of the assays dropped slightly for the agonist mode assays (BG1 from 93% to 90% and ER-bla from 81% to 79%). The specificity values showed large variations because the number of inactive reference chemicals available for these calculations is small (5). Only one reference chemical, progesterone, had inconclusive activity in the ER-bla antagonist mode assay. Progesterone was counted as a false positive in this analysis, which decreased the specificity of the assay from 100% to 80%.

### Concordance of qHTS data with ER binding assay results

The activities of the compounds in the two ER assays were compared with ER binding data^17^. The concordance between each qHTS ER assay and the ER binding assay is shown in Table 4. Both qHTS ER assays showed good concordance with the binding assay with the ER-bla assay performing better (91%) than the BG1 ER-luc assay (80%), with the ER-bla assay having more concordant negatives (93%) than the BG1 ER-luc assay (77%) and the BG1 ER-luc assay having more concordant positives (98%) than the ER-bla assay (76%).

### Identification of ER agonists

**Figure 2:**
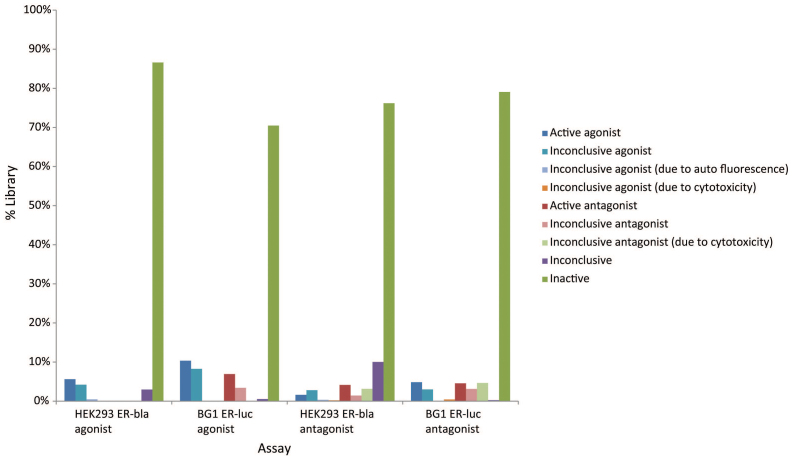
Activity outcome distribution of 10,496 (8,311 unique) compounds in the ER agonist and antagonist mode assays. Detailed activity outcome definitions can be found in Supplementary Tables S3 and S4.

**Figure 3:**
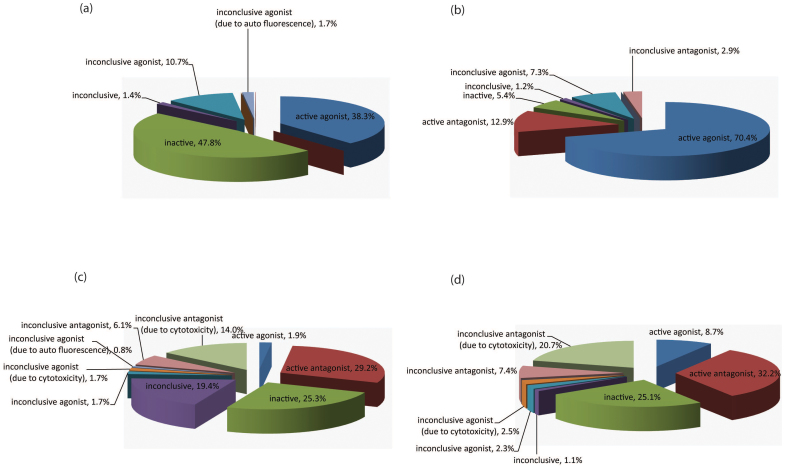
Activity distribution of (a) the active agonists identified by the BG1 ER-luc agonist mode assay in the ER-bla agonist mode assay; (b) the active agonists identified by the ER-bla agonist mode assay in the BG1 ER-luc agonist mode assay; (c) the active antagonists identified by the BG1 ER-luc antagonist mode assay in the ER-bla antagonist mode assay; and (d) the active antagonists identified by the ER-bla antagonist mode assay in the BG1 ER-luc antagonist mode assay.

All samples in the Tox21 10K collection were assigned one of the following activity outcome categories (see Supplementary Methods and Results for more detail): active agonist, inconclusive agonist (due to poor curve quality), inconclusive agonist (due to auto fluorescence), active antagonist, inconclusive antagonist (due to poor curve quality), inconclusive (activity direction could not be determined), or inactive. The antagonist outcome labels are for compounds showing inhibition in these assays that was not necessarily ER antagonism but rather might reflect increased cytotoxicity. The activity distributions for both the BG1 ER-luc and the ER-bla assays are shown in Figure 2 and Table 5. Approximately 5.6% of the library was identified as active agonists in the ER-bla assay, 86.6% as inactive and the rest of library (7.7%) were assigned one of the inconclusive activity categories. In comparison, the BG1 ER-luc assay identified nearly twice as many compounds (10.4% of the library) as active agonists and 70.5% of the library as inactive. Figure 3(a) shows the activity distribution of the active agonists identified from the BG1 ER-luc assay in the ER-bla assay. About half (48.9%) of the active agonists in the BG1 ER-luc assay were identified by the ER-bla assay as either active (38.3%) or inconclusive (10.7%) agonists, and the other half were nearly all as inactive (47.8%). On the other hand, most (77.7%) of the active agonists from the ER-bla assay were also identified as either active (70.4%) or inconclusive (7.3%) agonists in the BG1 ER-luc assay (Figure 3(b)). According to these results, it appears that the major difference between the two assays is that the ER-bla assay is less sensitive in detecting ER interacting compounds (Suppl. Figure 1), and the reason could be that the ER-bla assay has only the ER LBD whereas the BG1 ER-luc assay has the full length receptor. Overall, 4.0% of the library was identified as active agonists by both assays (12% by either) and 68.3% as inactive by both assays.

### Identification of ER antagonists

All samples in the Tox21 10K collection were assigned one of the following activity outcome categories in the BG1 ER-luc and the ER-bla antagonist mode assays (see Supplementary Methods and Results for more detail): active antagonist, inconclusive antagonist (due to poor curve quality), inconclusive antagonist (due to cytotoxicity), active agonist, inconclusive agonist (due to poor curve quality), inconclusive agonist (due to auto fluorescence), inconclusive agonist (due to cytotoxicity), inconclusive (activity direction could not be determined) or inactive. The agonist outcome labels are for compounds showing activation in these assays that was not necessarily ER agonism but rather compound auto fluorescence. The activity distributions for these two antagonist mode assays are shown in Figure 2 and Table 5. The ER-bla assay identified 4.1% of the library as active antagonists, 76.2% as inactive, 1.6% showing active activation, and the rest 18.0% as one of the inconclusive activity categories. Similar to the agonist mode assays, the BG1 ER-luc antagonist mode assay identified more actives than its ER-bla counter version, with 4.6% of the library identified as active antagonists, 79.1% inactive, and 16.3% inconclusive or showing active activation. In addition, the BG1 ER-luc assay identified a larger fraction of the library (4.7%) as inconclusive antagonist due to concurrent cytotoxicity than that identified by the ER-bla assay (3.2%). Of the active antagonists identified by the BG1 ER-luc assay (Figure 3(c)), about half (49.3%) were identified by the ER-bla assay as one of the antagonist categories, and the other half were either inactive (25.3%) or inconclusive (23.6%) in the ER-bla assay. The activity distribution of the active antagonists identified by the ER-bla assay in the BG1 ER-luc assay (Figure 3(d)) shows a slightly different pattern, with the majority (60.2%) identified as one of the antagonist categories, a similar fraction as inactive (25.1%), and a much smaller fraction as inconclusive (6.0%). From these results, the BG1 ER-luc assay again appeared to be more sensitive than the ER-bla assay but to a lesser extent than the agonist mode assays. Taken together, only 1.3% of the library was identified as active antagonists by both ER antagonist mode assays (7.4% by at least one of the assays) and 68.7% identified as inactive by both assays. Therefore, the agreement between the two antagonist mode ER assays appears to be lower than that between the two agonist mode assays.

### Structure classes of identified ER agonists and antagonists

**Figure 4:**
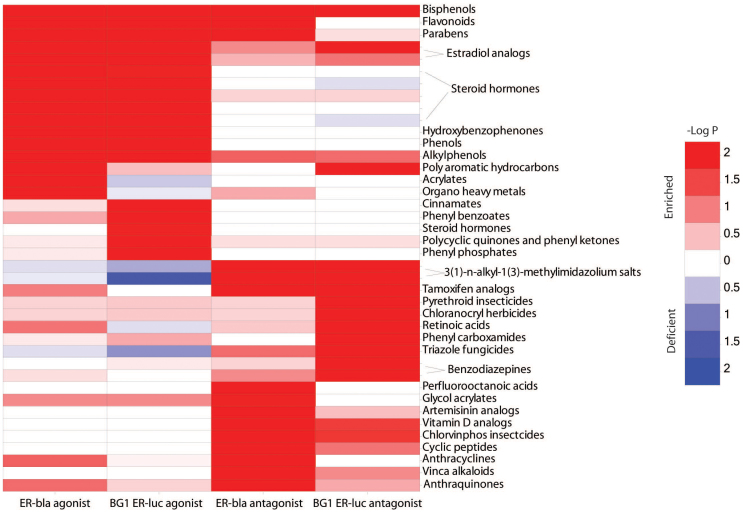
Example structure classes that are enriched with active agonists in the agonist mode ER assays or active antagonists in the antagonist mode ER assays. The heat map is colored by the significance (log p-value) of enrichment, where a darker red indicates a higher degree of enrichment and darker blue indicates a higher degree of deficiency of actives.

The 10K library was clustered based on structural similarity (512-bit ChemAxon fingerprints; ChemAxon Ltd., Cambridge, MA, USA) using the self-organizing map (SOM) algorithm^20^, resulting in 651 clusters. Each cluster was evaluated for its composition of compounds in different activity categories for each ER assay. We identified 66 clusters that are enriched (Fisher's exact test: p < 0.01) with ER actives (active agonists for agonist mode assays and active antagonists for antagonist mode assays) in at least one of the ER assays (Figure 4). Some of the antagonist clusters are also enriched with inconclusive antagonists due to apparent cytotoxicity. These clusters were excluded from further analysis. A close examination of the remaining clusters revealed structural classes that are well known ER agonists or antagonists such as tamoxifen analogs, bisphenols, flavonoids, parabens^21^, sex steroid hormones and analogs, hydroxybenzophenones and phenols (such as those used as UV filters in sunscreens^22^ and other natural phenolic compounds^23^,^24^).

Of the structure classes that are significantly enriched with active antagonists, only a few were high-confidence antagonists being active in both the ER-bla and BG1 ER-luc antagonist mode assays including the well-known ER antagonist tamoxifen and its structural analogs. The cluster of vitamin D analogs^25^,^26^ was also found enriched with active antagonists in both ER assays, such as paricalcitol, alfacalcidol, calcitriol, 24R,25-dihydroxyvitamin D3, and calcipotriene. The cluster of 3(1)-methyl-1(3)-alkyl imidazolium ionic liquids^27^,^28^ and the class of chlorvinphos insecticides (e.g., chlorfenvinphos, Z-tetrachlorvinphos, tetrachlorvinphos) are also structure classes that are significantly enriched with active antagonists in both ER-bla and BG1 ER-luc, but there is no previous report on their ER activity, while ionic detergents are typically cytotoxic. A number of structure classes are found enriched with active antagonists only in the BG1 ER-luc assay with no or fewer actives in the ER-bla assay. Examples include the clusters of pyrethroid insecticides, chloranocryl herbicides, retinoic acids, phenyl carboxamides, triazole fungicides, and benzodiazepines. We also identified structure classes that are significantly enriched with active antagonists in the ER-bla assay with fewer or no actives in the BG1 ER-luc assay. Examples of such compound classes include the perfluoroalkyl acids, artemisinin and its derivatives, DNA intercalating agents (cyclic peptides, anthracyclines, anthraquinones), vinca alkaloids, and glycol acrylates (see Supplementary Results for more detailed discussion on these compounds). Some of these structure classes contain compounds that have no previously reported ER activity. Given the confounding problems of interference due to cytotoxicity, fluorescence quenching, and luciferase inhibition, all of which could yield positive results in these assays, confidence in such compounds being true ER antagonists is greatly reduced. Orthogonal assays, such as the MCF-7 cell proliferation assay^11^, are required to confirm their activity as antagonists.

## Discussion

Efficient methods to identify chemicals of potential human health concern are needed to investigate the large number of chemicals with inadequate toxicological data^13^,^15^,^29^. To provide assurance to the public that chemicals have been adequately assessed, screening methods with high sensitivity (i.e., low false negative testing rates) and sufficient specificity (i.e., low false positive rates) are needed to identify compounds that will be more comprehensively tested using more resource-intensive test methods. The assays evaluated here have different receptor formats, reporter gene technologies and cell backgrounds, and yield reasonably good agreement in results. Understanding how and why these assays differ, though, is critical in developing a screening method acceptable to stakeholders and the public^15^,^30^. Moreover, the concentration-response curves generated in triplicate runs for the Tox21 10K library provided a rich and complex data set with the unique opportunity to test and compare various data analysis strategies for active identification^31^. Important to note is that all concentration-response data have been released in to the public domain^32^ for other computational scientists to apply their own algorithms for data interpretation.

The differences between the ER-bla and the BG1 ER-luc assays are reflected by the structure class activities shown in Figure 4, for example, the bisphenols, tamoxifen analogs, and sex hormones are enriched in ER agonists/antagonists in both the ER-bla and BG1 ER-luc assays, whereas the flavonoids and parabens are only enriched in ER antagonists in the ER-bla assay and not the BG1 ER-luc assay. Some of the flavonoids (e.g., genistein, biochanin A, apigenin) acted as antagonists in the ER-bla assay and as agonists in the BG1 ER-luc assay. This behavior may be explained by selective receptor modulator activity^33^. One aspect of nuclear receptor pharmacology that affects how compounds are assigned active agonist or active antagonist calls results from partial agonist behavior in the assays. A partial agonist achieves less than the maximum response of a full agonist (e.g., 17β-estradiol) even at maximal activity^34^. Such activity is believed to result from differences in the levels of required co-regulator proteins in different cell lines combined with compound-specific effects on inducing receptor conformational changes and subsequent co-regulator affinities^35^,^36^. This activity affects not only agonist response (e.g., efficacy), but will result in antagonist behavior when the full agonist used in an antagonist mode assay is displaced by a partial agonist. Thus, compounds may be seen as partial agonists in one cell line with weak or no antagonist activity or, conversely, as antagonists with weak or no agonist activity. An example of this is the osteoporosis drug raloxifene with activities in the BG1 cell line of an active antagonist and in the HEK293 cell line of a partial agonist (22% efficacy). *In vivo*, this drug acts as an estrogen agonist in bone and cardiovascular tissue but as an antagonist in breast and uterine tissue^37^. Such results reinforce the need for using multiple assay approaches in identifying estrogenic active compounds.

Of the 24 structure classes that are significantly enriched with active antagonists, nine structure classes (e.g., pyrethroids) are found enriched with active antagonists only in the BG1 ER-luc assay with no or fewer actives in the ER-bla assay. Previous reports on the ER activity of pyrethroids have been mixed. One study considered them to be estrogen-like chemicals that act through pathways other than direct ER binding, and may function as endocrine modulators in both wildlife and humans^38^. Another study found a lack of significant estrogenic or antiestrogenic activity of the pyrethroid insecticides in three *in vitro* assays based on classic ERα-mediated mechanisms, and indicated that they do not impact the classic ERα-mediated activation pathway *in vitro*^39^. A third study, however, suggested that the endocrine activities of the pyrethroid insecticides are from their metabolites and environmental degradation products^40^. On the other hand, we also identified structure classes that are significantly enriched with active antagonists in the ER-bla assay with fewer or no actives in the BG1 ER-luc assay. One example of such compound class is the perfluoroalkyl acids. Polyfluorinated chemicals are widely used as surfactants and surface protectors for paper and textile coatings, polishes, food packaging, and fire-retardant foams^41^. Perfluoroalkyl acids have been reported to show estrogen-like activity *in vivo* and *in vitro*, hypothesized through direct ER binding in a manner similar to bisphenol A and nonylphenol^42^,^43^. The structure classes identified with known ER activities can serve as validation for our ER assays. These data illustrate the ability of qHTS to identify possible novel ER active chemicals for further follow-up confirmatory research.

As compounds could interact with the ER signaling pathway without directly binding to the ER protein, these compounds could be detected by the reporter gene assays discussed in this study, which are functional ER assays, but show no binding affinity in the ER binding assay. We have identified a number of such compounds. Flavonoids can act as both agonists and antagonists of the human ER. While some of these compounds, such as genistein and apigenin, act by directly binding to the ER, certain flavonoids are known to elicit effects on estrogen signaling independent of direct receptor binding^33^. Flavone, 5,6-benzoflavone, and chrysin were identified as agonists in both the ER-bla and BG1 ER-luc assays but showed no activity in the ER binding assay. Flavone has been reported to show antiestrogenic activity mediated via the c-Jun N-terminal protein kinase pathway^33^. Both 5,6-benzoflavone^44^ and chrysin^45^ are known ligands of the aryl hydrocarbon receptor (AhR) and have been reported to activate ERα through cross-talk between the AhR and ER signaling pathways. Some flavonoids, including chrysin and flavone, can also act as aromatase inhibitors and interfere with estrogen signaling via inhibition of E2 synthesis^46^,^47^,^48^,^49^.

Conversely, we have also identified 36 compounds that showed some affinity to ER in the binding assay but showed no conclusive activity or were inactive in our reporter gene assays. These compounds were all classified as weak binders with binding affinities less than 1/100 of that of E2 (logRBA < −2)^50^. Triclosan has been recently characterized as an endocrine disruptor^51^,^52^ and was identified as an inconclusive antagonist in the ER reporter gene assays because of apparent cytotoxicity. The acaricide dicofol has been identified as a weak estrogen mimetic in a yeast-based gene transcription assay designed with the human ER^53^ and a MCF-7 cell proliferation assay^54^. Dicofol only showed inconclusive agonistic activity in our ER assays with very low potencies (>30 μM) and efficacies (<30%). The binding affinity of the fungicide maneb (logRBA = −2.46) was similar to that of triclosan (logRBA = −2.4) and was inactive in all of our reporter assays. No other report on the ER activity of maneb was found. These results show that these compounds are not bona fide false negatives, but the reporter assays we employed in qHTS may not be sensitive enough to conclusively identify very weak ER disruptors. However, at least one of the two qHTS reporter assays identified all except one of the weak and very weak ER active reference chemicals.

## Conclusions

This Tox21 project generated extensive data sets that can be used to inform the prioritization of a large collection of chemicals for potential interactions with the ER and, hence, potential for endocrine disruption given sufficient exposure. The use of two different cellular assays illustrates the importance of using multiple assay approaches to comprehensively identify active chemicals. Complex biology and the diversity in chemical structures (e.g., selective modulator activity, assay variability and sensitivity, metabolic capacity, specific assay interference, cytotoxicity) can make the use of a single assay problematic. The vast majority of the chemicals have been shown inactive and thus expected to have a very low likelihood of affecting estrogen receptor activity. The minority of chemicals identified as active or inconclusive are a manageable number of chemicals that can be readily studied in more detail for potential endocrine disrupting effects.

## Methods

### Tox21 chemical library

The Tox21 chemical library^16^ consists of compounds mostly procured from commercial sources by the EPA, NTP, and NCGC^55^, for a total of ~10,500 plated compound solutions consisting of 8,311 unique chemical substances including pesticides, industrial, food-use, and drugs. The main criteria for selection of the Tox21 compounds included, but were not limited to, known or perceived environmental hazards or exposure concerns, physicochemical properties indicating suitability for HTS (molecular weight, volatility, solubility, logP), commercial availability, and cost. In addition, the Tox21 Chemical Selection Group designated 88 diverse compounds in the Tox21 library to serve as internal controls (Suppl. Table 6) to assess assay reproducibility and examine positional plate effects: these were included as duplicates in all screening plates^19^.

### ERα reporter gene assays and qHTS

Two ERα reporter gene assays, HEK293 ER-bla and BG1 ER-luc, were run in both agonist and antagonist modes in a qHTS format. The GeneBLAzer® ERα-UAS-bla GripTite™ (HEK293 ERα-bla; Invitrogen, Carlsbad, CA, USA) cells comprise a mammalian one-hybrid system stably expressing a β-lactamase reporter gene under the control of the GAL4 DNA-binding site and a fusion protein consisting of the human ERα ligand-binding domain and the GAL4 DNA-binding domain. The BG1Luc4E2 (BG1 ER-luc) cell line was provided by Dr. Michael S. Denison (University of California at Davis, USA). BG1 (human ovarian carcinoma) cells were stably transfected with an estrogen-responsive luciferase reporter gene plasmid (pGudLuc7ere) containing the estrogen responsive element (ERE) and luciferase reporter gene^9^. To help differentiate true ER antagonists from cytotoxic compounds, cell viability was determined in the same well that ER antagonist activity was measured. A luminescence-based cell viability assay measuring intracellular ATP levels (Promega, Madison, WI, USA) was multiplexed with the HEK293 ER-bla assay and a fluorescence-based cell viability assay measuring conserved and constitutive protease activities within live cells (Promega) was multiplexed with the BG1 ER-luc assay. In the qHTS format, each compound was tested at 15 concentrations ranging from 1.1 nM to 92 μM. Detailed cell culture and qHTS assay conditions can be found on pages 2–4 and pages 6–7 in the Supplementary Information.

### Auto-fluorescence assay

An auto-fluorescence assay was performed to measure compound auto-fluorescence at three different wavelengths - green, blue, and red. The green and blue wavelengths are the same as the ones used for the HEK293 ER-bla assay. This assay was designed to filter out auto-fluorescence-induced assay artifacts. Detailed cell culture and qHTS conditions for this assay can be found in the Supplementary Information.

### qHTS data analysis

Detailed analysis methods for concentration-response data generated from the triplicate runs and assigning compounds into different activity categories can be found in the Supplementary Information. All concentration-response data have been released to the public domain (PubChem assay IDs: 743075, 743077, 743069, 743078, 743074, 743079, 743080, 743091, 743081)^56^.

### ER reference chemicals

To evaluate the qHTS ER assay results, compound activity outcomes were compared to a set of 39 ER reference chemicals (all of which are in the Tox21 10K library) from the EDSP21 working-group, obtained by expert review of the literature and NTP Interagency Center for the Evaluation of Alternative Toxicological Methods (NICEATM) consensus. These chemicals have been used to validate ER *in vitro* assays and were taken from the Organization for Economic Cooperation and Development (OECD) Test Guideline (TG) 457 BG1 guidance document^57^. Reference chemicals were classified for expected ER activity as strong (3), strong-moderate (1), moderate (11, 6 of which are antagonists), weak (15), very weak/agonist (metabolism required) (4), or negative (5). These chemicals as well as their activity outcomes in the qHTS ER assays are listed in Suppl. Table 5.

### Comparison of qHTS data with ER binding assay results

ER binding data are available^17^ for 592 compounds (unique CAS numbers) in the Tox21 10K library. The activities of these compounds in the two ER assays in both agonist and antagonist modes were compared with the binding results, where both active agonists and antagonists in the ER qHTS assays were considered “active” and all inconclusive compounds (see Activity Assignments section in Supplementary Information) were excluded from the comparison. To compare each qHTS assay with the binding assay, compounds that were active in both the qHTS assay and the binding assay [compounds with binding affinities less than 1/1000 of that of E2 (logRBA < −3) were considered inactive in the binding assay^17^] were counted as concordant positives (CP), compounds that were active only in the qHTS assay or the binding assay were counted as discordant positives (DP) or discordant negatives (DN), respectively, and compounds that were inactive in both assays were counted as concordant negatives (CN). The overall concordance ((CP + CN)/(CP + DP + CN + DN)) between each qHTS ER assay and the ER binding assay was then calculated.

## Author Contributions

M.X., R.H., R.R.T., R.J.K. and C.P.A. designed the study. S.S. performed the experiments and collected data. R.H. performed statistical analysis of all data. R.H., M.X., R.R.T. and K.A.H. wrote the manuscript. M.T.M., D.M.R., R.S.J., K.A.H., W.C., J.-H.H., K.R.S., P.C., J.F., W.T., D.M.R. and T.Z. aided data analysis. D.J.D., K.A.H., K.L.W., A.S. and W.T. aided study design. P.S. plated the compound library and helped with screening design. All authors reviewed the manuscript.

## Supplementary Material

Supplementary Information Supplementary Information

## Figures and Tables

**Table 1:** qHTS assay summary statistics†

Assay	S/B*	CV (%)*	Z' factor	Positive Control	Positive Control AC50 (M) (±fold)
HEK293 ER-bla agonist	4.6 ± 0.6	4.7 ± 3.7	0.53 ± 0.09	17 β-Estradiol	3.14 × 10^−10^ (1.4)
HEK293 ER-bla antagonist	3.3 ± 0.8	5.1 ± 2.8	0.41 ± 0.10	Tamoxifen	5.01 × 10^−9^ (1.4)
HEK293 ER-bla viability	132.6 ± 8.2	9.4 ± 2.5	0.76 ± 0.06	Tetra n-octyl ammonium bromide	N/A
BG1 ER-luc agonist	2.5 ± 0.3	10.3 ± 4.6	0.50 ± 0.25	17 β-Estradiol	2.74 × 10^−11^ (2.8)
BG1 ER-luc antagonist	8.0 ± 0.9	6.5 ± 2.8	0.77 ± 0.07	Tamoxifen	7.30 × 10^−8^ (1.1)
BG1 ER-luc viability	6.1 ± 0.9	7.2 ± 2.1	0.81 ± 0.06	Tetra n-octyl ammonium bromide	N/A

^†^Data presented as mean ± standard deviation.

*S/B = Signal to Background, CV = Coefficient of Variance, AC50 = Concentration at 50% activity.

**Table 2:** Assay performances measured by reproducibility of the triplicate runs and the Tox21-88 duplicates

Assay	Active Match	Inactive Match	Inconclusive	Mismatch	AC50 fold change
Triplicate Runs					
HEK293 ER-bla agonist	7.01%	87.11%	5.87%	0.01%	1.36
HEK293 ER-bla antagonist	9.84%	77.86%	11.95%	0.34%	1.49
HEK293 ER-bla viability	3.59%	90.69%	5.66%	0.06%	1.45
BG1 ER-luc agonist	16.43%	71.22%	12.05%	0.28%	1.52
BG1 ER-luc antagonist	12.03%	79.72%	7.96%	0.29%	1.48
BG1 ER-luc viability	5.86%	88.57%	5.56%	0.01%	1.37
Tox21-88					
HEK293 ER-bla agonist	13.01%	74.38%	11.70%	0.91%	1.22
HEK293 ER-bla antagonist	22.44%	55.17%	21.14%	1.25%	1.39
HEK293 ER-bla viability	4.26%	80.91%	13.92%	0.91%	1.47
BG1 ER-luc agonist	27.22%	51.93%	18.30%	2.56%	1.43
BG1 ER-luc antagonist	19.03%	66.59%	13.47%	0.91%	1.31
BG1 ER-luc viability	9.55%	79.15%	11.02%	0.28%	1.23

**Table 3:** Assay reliability measured by accuracy in identifying known ER actives as defined by results obtained in other *in vitro* and *in vivo* assays (values shown in parentheses included compounds with inconclusive activity outcomes)

Assay	FP	FN	TP	TN	Specificity	Sensitivity	Accuracy	p-value*
HEK293 ER-bla agonist	0 (2)	5 (6)	19 (28)	3 (3)	100% (60%)	79% (82%)	81% (79%)	1.9 × 10^−2^ (7.0 × 10^−2^)
BG1 ER-luc agonist	1 (3)	1 (1)	26 (33)	2 (2)	67% (40%)	96% (97%)	93% (90%)	2.0 × 10^−2^ (3.8 × 10^−2^)
HEK293 ER-bla antagonist	0 (1)	0 (0)	6 (6)	4 (4)	100% (80%)	100% (100%)	100% (91%)	5.0 × 10^−3^ (1.5 × 10^−2^)
BG1 ER-luc antagonist	1 (1)	0 (0)	6 (6)	4 (4)	80% (80%)	100% (100%)	91% (91%)	1.5 × 10^−2^ (1.5 × 10^−2^)

*Fisher's exact test; TP = True Positive, FP = False Positive, TN = True Negative, FN = False Negative.

**Table 4:** Performance of the ER qHTS assays in identifying potential ER interacting compounds as measured by results in an ER binding assay

Assay	DP	DN	CP	CN	Inconclusive	Concordance	p-value*
HEK293 ER-bla	24	13	41	345	169	91%	2.7 × 10^−17^
BG1 ER-luc	74	1	54	254	209	80%	2.8 × 10^−10^

*Fisher's exact test; CP = Concordant Positive, DP = Discordant Positive, CN = Concordant Negative, DN = Discordant Negative.

**Table 5:** Activity outcome distribution of 10,496 (8,311 unique) compounds in the ER agonist and antagonist mode assays

Activity outcome	HEK293 ER-bla agonist	BG1 ER-luc agonist	HEK293 ER-bla antagonist	BG1 ER-luc antagonist
Active agonist	5.63%	10.36%	1.62%	4.86%
Inconclusive agonist	4.23%	8.29%	2.82%	3.00%
Inconclusive agonist (due to cytotoxicity)	0.00%	0.00%	0.24%	0.44%
Inconclusive agonist (due to auto fluorescence)	0.47%	0.00%	0.35%	0.00%
Active antagonist	0.00%	6.94%	4.14%	4.56%
Inconclusive antagonist	0.09%	3.41%	1.45%	3.13%
Inconclusive antagonist (due to cytotoxicity)	0.00%	0.00%	3.15%	4.67%
Inconclusive	2.96%	0.53%	10.02%	0.26%
Inactive	86.62%	70.47%	76.20%	79.09%
