# Supplementary material for: Profiling of the Tox21 10K compound library for agonists and antagonists of the estrogen receptor alpha signaling pathway
Source: Sci Rep. 2014 Jul 11;4:5664. doi: 10.1038/srep05664 (PMC4092345; doi:10.1038/srep05664)
Supplement: Supplementary Information [file srep05664-s1.pdf]

Supplemental Material

Profiling of the Tox21 10K compound library for environmental agonists and antagonists of the estrogen receptor signaling pathway

Ruili Huang, Srilatha Sakamuru, Matt T. Martin, David M. Reif, Richard S. Judson, Keith A. Houck, Warren Casey, Jui-Hua Hsieh, Keith Shockley, Patricia Ceger, Jennifer Fostel, Kristine L. Witt, Weida Tong, Daniel M. Rotroff, Tongan Zhao, Paul Shinn, Anton Simeonov, David J. Dix, Christopher P. Austin, Robert J Kavlock, Raymond R. Tice, Menghang Xia

Materials and Methods

Cell culture. The GeneBLAzer® ERα-UAS-bla GripTite™ (HEK293 ER-bla) cells obtained from Invitrogen (Carlsbad, CA, USA) is a mammalian one-hybrid system stably expressing a β-lactamase reporter gene under the control of the GAL4 DNA-binding site and a fusion protein consisting of the human ERα ligand-binding domain and the GAL4 DNA-binding domain. HEK293 ER-bla cells were cultured in DMEM (Dulbecco's Modified Eagle Medium) high-glucose (Invitrogen) supplemented with 10% dialyzed fetal bovine serum (FBS), 100 µg/mL zeocin, 80 µg/mL hygromycin, and 100 U/mL penicillin/100 µg/mL streptomycin (Invitrogen). The BG1Luc4E2 (BG1 ER-luc) cell line was provided by Dr. Michael S. Denison (University of California at Davis, USA). BG1 (human ovarian carcinoma) cells were stably transfected with an estrogen responsive luciferase reporter gene plasmid (pGudLuc7ere) containing the estrogen responsive element (ERE) and luciferase reporter gene. BG1 ER-luc cells were cultured in MEMα Medium (Invitrogen) supplemented with 10% FBS, 400 µg/mL G418 and 100 U/mL penicillin/100 µg/mL streptomycin. The HEK293 (wild-type) cells from ATCC (Manassas, VA) were cultured in DMEM medium containing 10% FBS and 100 U/mL penicillin/100 µg/mL streptomycin (Invitrogen). All the cells were maintained at 37ºC under a humidified atmosphere and 5% CO2.

qHTS of ERα beta-lactamase and BG1 ER luciferase reporter gene assays. The HEK293 ER-bla cells were cultured in assay medium (DMEM phenol red free medium containing 2% charcoal stripped FBS) overnight and the BG1 ER-Luc cells were in assay medium (DMEM phenol red free medium containing 10% charcoal stripped FBS) for 5 days prior to the screening. Both agonist and antagonist screen protocols were summarized in **Table S1 and Table S2**.

For agonist mode screening, HEK293 ER-bla cells suspended in assay medium were dispensed at 5,000 cells/6 µL/well in 1536-well black wall/clear bottom plates (Greiner Bio-One North America, Monroe, NC, USA) and BG1Luc4E2 cells dispensed at 4,000 BG1 ER-Luc cells/5 µL/well in 1536-well white tissue cultured plates (Greiner Bio-One) using a Thermo Scientific Multidrop Combi (Thermo Fisher Scientific Inc., Waltham,

MA, USA). After the assay plates were incubated at 37ºC for 5 h (HEK293 ER-bla cells) or 24 h (BG1 ER-Luc cells), 23 nL of compounds dissolved in dimethyl sulfoxide (DMSO), positive controls, or DMSO was transferred to the assay plates by a pintool (Kalypsys, San Diego, CA, USA). The final compound concentration in the 5 µL assay volume ranged from 1 nM to 92 µM and in the 6 µL assay volume ranged from 0.8 nM to 76 µM with √5-fold dilution for 15 concentrations. For HEK293 ER-bla screening, the plate format of positive control was as follows: Column 1, concentration-response titration of β-estradiol (CASRN 50-28-2) (Sigma-Aldrich, St. Louis, MO, USA) from 0.1 pM to 3.83 µM; Column 2, β-estradiol at 40 nM; Column 3, β-estradiol at 20 nM; Column 4, DMSO only. The plates were incubated at 37°C for 18 hr. After 1 µL of LiveBLAzer™ B/G FRET substrate (Invitrogen) was added using a Flying Reagent Dispenser (FRD, Aurora Discovery, Carlsbad, CA, USA), the plates were incubated at room temperature (RT) for 2 h, and fluorescence intensity at 460 and 530 nm emission was measured at 405 nm excitation by an Envision plate reader (Perkin Elmer, Shelton, CT, USA). For BG1 ER-Luc screening, the plate format of positive control was as follows: Column 1, concentration-response titration of β-estradiol (Sigma-Aldrich) from 0.001 pM to 46 nM; Column 2, β-estradiol in top 16 wells at 10 nM and bottom 16 wells at 5 nM; Column 3 & 4, β-estradiol in top 16 wells at 2.5 µM and 0.5 µM respectively; Column 3 & 4, DMSO only in bottom 16 wells. The assay plates of BG1 ER-Luc screen were incubated at 37°C for 22 hr. After 5 µL of ONE-Glo™ Luciferase Assay reagent (Promega, Madison, WI, USA) was added using an FRD (Aurora Discovery), the plates were incubated at RT for 30 min, and luminescence intensity was measured by ViewLux plate reader (Perkin Elmer). Data was expressed as the ratio of 460 nm/530 nm emissions for HEK293 ER-bla assay and expressed as relative luminescence units for BG1 ER-Luc assay. For primary data analysis, raw plate reads for each titration point were first normalized relative to β-estradiol control (10 nM for the BG1 ER-Luc assay and 40 nM for the HEK293 ER-bla assay, 100%) and DMSO only wells (basal, 0%).

For antagonist mode screening, the assays were run multiplexed with a cell viability assay, HEK293 ER-bla cells suspended in assay medium were dispensed at 5,000 cells/5 µL/well in 1536-well black wall/clear bottom plates (Greiner Bio-One) and BG1Luc4E2

cells dispensed at 4,000 BG1 ER-Luc cells/4 µL/well in 1536-well white tissue cultured plates (Greiner Bio-One) using a Thermo Scientific Multidrop Combi (Thermo Fisher Scientific Inc.). After assay plates were incubated at 37ºC for 5 h (HEK293 ER-bla assay) or 24 h (BG1 ER-Luc assay), 23 nL of compounds dissolved in DMSO, positive controls, or DMSO was transferred to the assay plate by a pintool (Kalypsys), followed by the addition of 0.5 nM of β-estradiol (stimulator) on the top. The plate format of positive control was as follows: Column 1, concentration-response titration of 4-hydroxy tamoxifen (CASRN 68047-06-3) (Sigma-Aldrich) from 0.015 nM to 1 µM; Column 2, 1 µM of 4-hydroxy tamoxifen with 0.5 nM β-estradiol in top 16 wells and bottom 16 wells for 92 µM of tetraoctyl ammonium bromide (CASRN 14866-33-2) (Sigma-Aldrich) with 0.5 nM β-estradiol; Column 3 and 4, DMSO only with 0.5 nM β-estradiol in the top 16 wells; Column 3 and 4, DMSO only with assay buffer in the bottom 16 wells. The plates were incubated at 37°C for 18 h (HEK293 ER-bla assay) or 22 h (BG1 ER-Luc assay). For HEK293 ER-bla assay, 1 µL of LiveBLAzer™ B/G FRET substrate (Invitrogen) was added into each well using an FRD Dispenser (Aurora Discovery). After the assay plates were incubated for 2 h at RT, the fluorescence intensity at 460 and 530 nm emissions was measured at 405 nm excitation by an Envision plate reader (Perkin Elmer), followed by an addition of 4 µL/well of cell viability reagent (CellTiter-Glo, Promega) using a FRD (Aurora Discovery). After 30 min incubation at RT, the luminescence intensity in the plates was measured using a ViewLux plate reader (Perkin Elmer). For BG1 ER-Luc assay, 1 µL/well of CellTiter-Fluor reagent (Promega) was added into the assay plates using an FRD (Aurora Discovery). After 30 min incubation at 37ºC, the fluorescence intensity in the plates was measured using a ViewLux plate reader (PerkinElmer), followed by the addition of 4 µL of ONE-Glo™ Luciferase Assay reagent (Promega) using an FRD (Aurora Discovery), the plates were incubated at RT for 30 min, and luminescence intensity was measured by ViewLux plate reader (Perkin Elmer). Data was expressed as the ratio of 460 nm/530 nm emissions for HEK293 ER-bla assay and expressed as relative luminescence units for BG1Luc4E2 assay. For primary data analysis, raw plate reads for each titration point were first normalized relative to 0.5 nM β-estradiol in presence of 1 µM 4-hydroxy tamoxifen (ER antagonist mode) or tetra n-

octyl ammonium bromide (cell viability) control (-100%) and 0.5 nM β-estradiol (control, 0%).

Auto-fluorescence assay. The auto-fluorescence assay was performed in the cells or cell free assay medium. The cells or assay medium only were dispensed at 2,000 cells/5 µL/well or at 5 µL assay medium/well in 1536-well black wall/clear bottom plates (Greiner Bio-One) using a Scientific Multidrop Combi (Thermo Fisher Scientific Inc.). After the assay plates were incubated at a 37ºC/5% CO2 incubator for 5 hr, 23 nL of compounds dissolved in DMSO, positive controls, or DMSO was transferred to the assay plates by a pintool (Kalypsys). The final compound concentration in the 5 µL assay volume ranged from 5 nM to 92 µM √5-fold dilution for 15 concentrations. The plate format of positive control was as follows: concentration-response titration of fluorescein (CASRN 2321-07-5) (Sigma-Aldrich) (4.5 nM to 10.0 µM) and 0.6 µM fluorescein in the top halves of column 1 and 2, respectively; concentration-response titration of triamterene (CASRN 396-01-0) (Sigma-Aldrich) (45 nM to 100.0 µM) and 20 µM triamterene in the bottom halves of column 1 and 2, respectively; concentration-response titration of Rose Bengal (CASRN 632-69-9) (Sigma-Aldrich) (45.0 nM to 100.0 µM) and 50 µM Rose Bengal in the top halves of column 3 and 4, respectively; the bottom halves of column 3 and 4 have DMSO only. The plates were incubated at 37°C for 16 hr. The fluorescence intensities (485 nm excitation/535 nm emission for green fluorescence; 405 nm excitation/460 nm emission for blue fluorescence; 540 nm excitation/690 nm emission for red fluorescence) were measured by an Envision plate reader (Perkin Elmer). For primary data analysis, raw plate reads for each titration point were first normalized relative to fluorescein control (0.6 µM, 100%) and DMSO only wells (basal, 0%) for green fluorescence, triamterene control (20 µM, 100%) and DMSO only wells (basal, 0%) for blue fluorescence, and Rose Bengal control (50 µM, 100%) and DMSO only wells (basal, 0%) for red fluorescence.

qHTS Data Analysis. Analysis of compound concentration–response data was performed as previously described[1]. Briefly, raw plate reads for each titration point were first normalized relative to the positive control compound (agonist mode: 100%; antagonist

mode: 0%) and DMSO-only wells (agonist mode: 0%; antagonist mode: -100%) as follows: % Activity = $((V_{compound} – V_{DMSO})/(V_{pos} – V_{DMSO})) \times 100$, where $V_{compound}$ denotes the compound well values, V_{pos} denotes the median value of the positive control wells, and V_{DMSO} denotes the median values of the DMSO-only wells, and then corrected by applying a NCGC in-house pattern correction algorithm using compound-free control plates (i.e., DMSO-only plates) at the beginning and end of the compound plate stack. Concentration–response titration points for each compound were fitted to a four-parameter Hill equation[2] yielding concentrations of half-maximal activity (AC_{50}) and maximal response (efficacy) values. Compounds were designated as Class 1–5 according to the type of concentration–response curve observed[1,3]. Curve classes are heuristic measures of data confidence, classifying concentration–responses on the basis of efficacy, the number of data points observed above background activity, and the quality of fit.

Activity assignments based on triplicate run. Each curve class was first converted to a curve rank as previously described[1] such that more potent and efficacious compounds with higher quality curves were assigned a higher rank. Curve ranks should be viewed as qualitative descriptors of the concentration response activity of the compound. Curve ranks from replicate runs of a compound were averaged, and the activity outcome of each compound in the BG1 ER-luc assays and from each readout of the HEK293 ER-bla assays (ratio, 460 nm, 530 nm and cell viability) was assigned based on its average curve rank and reproducibility call as shown in Table S3. The final activity outcome of each compound was determined based on its multi channel readout activity as shown in Table S4. For antagonist mode assays, the cell viability counter screen data were used to flag potential cytotoxic artifacts. For the HEK293 ER-bla agonist mode assay, potential artifacts produced by blue fluorescent compounds were flagged using both the compound auto fluorescence profiling data and promiscuous compound activity shown in the 460 nm readout of all the additional BLA assays screened in Tox21 with this compound library.

Reproducibility calls. Samples were first assigned an activity outcome based on their curve class as follows: inactive (class 4), active agonist/antagonist (class 1.1, 2.1; class 5 due to super potency (AC50<lowest test concentration)), agonist/antagonist (class 1.2, 2.2), inconclusive agonist/antagonist (all other non-5 classes), no call (other cases of class 5). Each activity outcome category (excluding the "no call" category, which was treated as missing data) was then assigned a score: active agonist (3), agonist (2), inconclusive agonist (1), active antagonist (-3), antagonist (-2), inconclusive antagonist (-1), inactive (0). The pair-wise activity outcome score differences for all replicates of each sample were then averaged and the % of inactive calls for the sample calculated to determine the final reproducibility call of the sample: active match (average score difference <1.1, %inactive call <25%), inactive match (average score difference <1.1, %inactive call >50%), mismatch (average score difference >2.5), inconclusive (all other cases).

Identification of auto fluorescence (HEK293 ER-bla agonist mode assay) and cytotoxicity (antagonist mode ER assays) artifacts. In the HEK293 ER-bla agonist mode assay, blue fluorescent compounds could show the same phenotype as agonists. Two approaches were used to identify potential auto fluorescent artifacts to distinguish them from true ER agonists. One approach is the auto fluorescence detection counter screen measured at 460 nm (blue) in the ER background HEK293 cells and cell free medium. Any sample with the agonist phenotype in the blue channel of the BLA assay that also showed activation in the auto fluorescence counter screen with an AC50 difference <3 fold was identified as a potential auto fluorescent false positive. This approach identified 34 such samples. The second approach is examining the activity of each sample in all the eight BLA assays screened to date in Tox21 10K library. Samples with the agonist phenotype in the blue channel of the HEK293 ER-bla assay that also had an >4 average curve rank in the blue channel of all the BLA assays were considered promiscuously active in BLA assays and potentially blue fluorescent. This second approach identified 39 potential fluorescent false positives, 24 of which overlapped with those identified by the first approach. The two approaches yielded a total of 49 unique samples that were assigned the "inconclusive agonist (fluorescent)" activity outcome category, most of which are known blue fluorescent compounds (see PubChem[4] assay ID 743077).

The BG1 ER-luc assay has a luminescence- based readout so that interference from compound auto fluorescence was not a concern for this assay. The activity outcomes of the 49 samples identified as auto fluorescent in the HEK293 ER-bla assay were examined in the BG1 ER-luc assay and 19 of them were assigned the "active agonist" category, the ER activity of which could be, therefore, real and not artificial, and the rest of the 49 samples were either inactive or inconclusive in the BG1 ER-luc assay.

As cytotoxic compounds could show the same inhibitory phenotype as antagonists in these assays, we need an effective strategy to distinguish true ER antagonists from a cytotoxicity-related false positive response. For this reason, each antagonist mode assay was accompanied with a cell viability readout that serves as the counter screen. As an alternative to the cell viability counter screen, the control channel (530 nm readout) of the BLA assay could be used to identify potential cytotoxic compounds. Either activation or inhibition shown in this channel could be an indication of cytotoxicity[1]. To compare the effectiveness of the 530 nm readout and the cell viability counter screen at identifying potential cytotoxicity artifacts, activity outcomes were assigned to the screened samples using both approaches (Table S4(b)) and the set of known ER reference compounds was used to evaluate the accuracy of these assignments. Both approaches achieved 100% specificity in correctly identifying the reference compounds. Filtering with the cell viability counter screen resulted in better sensitivity compared to the 530 nm readout for both all reference compounds (59% vs. 50%) and when only the known ER antagonists were used (100% vs. 70%) in the evaluation. Three known ER antagonists were misclassified as cytotoxicity artifacts using the 530 nm readout resulting in lower sensitivity.

*Structure classes of identified ER agonists and antagonists – partial vs. full-length receptor cell lines***.** Other hydroxylated aromatic hydrocarbons that co-cluster with the phenols have been reported to show estrogenic activities, such as 2- and 3-hydroxyfluorenes and 1-hydroxypyrene[5], which are commonly found in cigarette smoke condensate[6] and identified as active agonists in both the HEK293 ER-bla and BG1 ER-

luc assays. Alkylphenols, such as nonylphenols, are precursors to commercially important detergents and are produced in large volumes annually[7]. Nonylphenols and other industrial alkylphenols have been known as synthetic environmental estrogens with generally weak estrogenic activities reported[8-10]. The cluster of alkylphenols, including nonylphenols, in the 10K library was found enriched with active agonists in both of the ER agonist mode assays with some compounds also acting as antagonists in the ER antagonist mode assays.

Polycyclic aromatic hydrocarbons (PAHs) are a large group of environmental and dietary toxicants, which and/or the metabolites of which have been reported to show either estrogenic or antiestrogenic activities[11-14]. Our cluster of PAHs showed an enrichment of active agonists in the ER agonist mode assays (more so in the HEK293 ER-bla assay) and enrichment of active antagonists in the BG1 ER-luc antagonist mode assay. Of particular note was the PAH 7,12-dimethylbenz(a)anthracene, which acted as an agonist in the BG1 ER-luc agonist mode assay and as an antagonist in the corresponding antagonist mode assay at low concentrations (<1 µM) but started to act like an agonist at higher concentrations. These compounds are reported to require reduction by cytochrome P450 monooxygenase to generate phenolic groups. These compounds are usually reported as negative in ER binding assays lacking metabolism. Activity in the assays reported here may indicate some metabolic capacity of the cell lines used. A similar class of compounds, the polycyclic quinones and phenyl ketones[15], on the other hand, showed a significant enrichment of active agonists in the BG1 ER-luc assay in both agonist and antagonist modes, but less so in the HEK293 ER-bla assay. No ER activity has been reported previously for some compounds in this class (e.g., dibenzosuberone, which was active in both the BG1 ER-luc and HEK293 ER-bla assays). Organophosphates commonly used as flame retardants is another class of compounds that only showed enrichment in active agonists in the BG1 ER-luc assay and not the HEK293 ER-bla assay. The ER activities of some of these compounds (e.g., triphenyl phosphate), which was also identified by the HEK293 ER-bla assay as an inconclusive agonist, have been reported recently[16].

The trimethylolpropane trimethacrylate class of compounds is a monomer used in the manufacture of acrylics and plastic components in a wide variety of products. These chemicals used as dental materials have been tested for estrogenic activity *in vitro* but with negative results[17]. This acrylate class of compounds, however, showed an enrichment of active agonists in our HEK293 ER-bla agonist mode assay. These apparent ER agonists include acrylates such as trimethylolpropane triacrylate, pentaerythritol triacrylate, dipentaerythritol pentaacrylate, and pentaerythritol tetraacrylate. These compounds also acted as agonists in the BG1 ER-luc antagonist mode at concentrations <30 µM, but the signals started to drop at higher concentrations perhaps due to cytotoxicity. Some of these compounds acted in a similar fashion in the HEK293 ER-bla antagonist mode and BG1 ER-luc agonist mode assays as well with some ER activation shown at low concentrations and inhibition due to cytotoxicity at high concentrations. There is a group of organometalic compounds in the 10K library including triphenyltin, triphenyllead, triphenylbismuth, and phenylmercuric compounds that showed an enrichment of active agonists in the HEK293 ER-bla agonist mode assay. Triphenylbismuth also acted as an agonist in the BG1 ER-luc assay. Organotin compounds have recently been reported to inhibit the transcriptional activation of human ER[18]. No report has been found on the estrogenic activity of the other organometalic compounds. These organometalic compounds showed inhibition activity in the antagonist mode HEK293 ER-bla assay and the BG1 ER-luc assays but were mostly classified as inconclusive antagonists due to cytotoxicity. Although no clear indication of ER activity has been reported for the class of cinnamates, commonly used in sunscreens and cosmetics[19], this structure class showed an enrichment of active agonists only in the BG1 ER-luc assay. Some of the cinnamates, such as 3-methylbutyl cinnamate and 2-phenylethyl 3-phenylprop-2-enoate, acted as agonists in the antagonist mode BG1 ER-luc assay as well. Negative to weak ER binding activities have been reported for compounds belong to the class of phenyl benzoates including phenyl parabens, phenyl phthalates, and phenyl salicylates, which are also common ingredients of cosmetics and sunscreens[20,21]. Similar to the cinnamates, we found this class of compounds enriched with active agonists in the BG1 ER-luc assay with most showing agonist activity also in the

antagonist mode assay. Phenyl 4-aminosalicylate and phenylparaben were also found active in the HEK293 ER-bla agonist mode assay.

The cluster of chloranocryl herbicides was also enriched with active antagonists (e.g., cypromid, chloranocryl, propanil) only in the BG1 ER-luc assay. Of these herbicides, only propanil has been previously reported to show endocrine disruption but not through direct ERα binding[22]. No previous report of ER activity has been found on the other herbicides in this cluster. The well known histone deacetylase (HDAC) inhibitor, suberoylanilide hydroxamic acid (SAHA or vorinostat), was closely clustered with the chloranocryl herbicides and identified as an active antagonist in the BG1 ER-luc assay. SAHA has been reported to induce ERα degradation in the breast cancer MCF-7 cells via the C-terminal Hsc70 interacting protein-mediated ubiquitin pathway[23-26], which may explain its ER antagonist activity observed. The retinoic acids acted as active antagonists in BG1 ER-luc assay, but as active agonists in HEK293 ER-bla assay. Retinoic acid has been found previously to inhibit ER activity not through direct ER-binding but by altering the amount of ER protein bound to the ERE or affecting the transcriptional efficiency of this complex[27]. Another study found that retinoic acids decreased ERα expression in estrogen-responsive endometrial cancer cells[28]. The phenyl carboxamides are in one of the neighboring clusters of the retinoic acids. This cluster was enriched with active antagonists in the BG1 ER-luc assay but also contain some active agonists. The active antagonists in this class of compounds include retinoic acid receptor subtype-specific ligands, retinoid analogs AM580[29,30] and tamibarotene (Am80)[31]. No specific ER activity has been reported previously on these compounds but the structure activity analyses suggest that they may act in a fashion similar to the retinoic acids. The cluster of triazole fungicides was another structure class enriched in active antagonists (e.g., metconazole, diniconazole, penconazole, tebuconazole, myclobutanil, propiconazole, epoxiconazole, hexaconazole, fenbuconazole, tetraconazole, ipconazole) in the BG1 ER-luc assay with fewer actives in the HEK293 ER-bla assay. Of these compounds, only myclobutanil has been reported to bind to ERα and have antiestrogenic effect[32]. The benzodiazepine class of psychoactive drugs was also found enriched with active antagonists in the BG1 ER-luc assay and vatalanib, a tyrosine kinase inhibitor closely

clustered with the benzodiazepines, was identified as an active antagonist in both the BG1 ER-luc and the HEK293 ER-bla assay. However, none of these compounds has been reported previously to have ER activity.

Artemisinin and its derivatives are used as standard treatments for malaria[33]. The class of artemisinin analogs was also one of the classes found enriched with active antagonists only in the HEK293 ER-bla assay. Consistent with this observed antiestrogenic activity, artemisinin has been reported to selectively decrease functional levels of ERα and ablate estrogen-induced proliferation in human breast cancer cells[34]. No direct ER binding was reported, however. The class of DNA intercalating agents, enriched with active antagonists in the HEK293 ER-bla assay, showed interesting activities in both the HEK293 ER-bla and BG1 ER-luc assays. Rubitecan and actinomycin D were identified as active antagonists in both HEK293 ER-bla and BG1 ER-luc. Other compounds, such as daunorubicin, idarubicin, carminomycin, aclarubicin, plicamycin, chromomycin A3, adriamycin, and daunomycin, all acted as active antagonists in the HEK293 ER-bla assay, but in the BG1 ER-luc assay, some acted as antagonists and others acted as agonists. The only previous report related to their ER activity investigated actinomycin D, which was found to prevent the nuclear processing of ER[35]. A more recent report related to the ER activity of DNA intercalators was about XR5944, a compound not in the Tox21 10K library. XR5944 was reported to specifically inhibit the binding of ER to its consensus DNA sequence and its subsequent activity[36]. The DNA intercalating agents with apparent ER antagonist activity in our ER assays are likely to exert their activity in a similar fashion. Vinca alkaloids, including the antimicrotubule agents vinblastine and vincristine, were also found enriched with active antagonists in HEK293 ER-bla assay and less so in BG1 ER-luc assay. Vinca alkaloids have been reported to decrease ERα protein levels in the human breast cancer cell line MCF-7 and inhibit estradiol mediated transactivation at ERE-driven promoters[37]. Again, no direct ER binding was indicated. Finally, the class of glycol acrylates, which has wide industrial applications such as adhesives, solvents, coating materials and cosmetics, was found enriched with active antagonists in HEK293 ER-bla assay with some compounds acted as active agonists (e.g.,

ethylene acrylate) in both ER assays. However, no previous report has been found on this class of compounds exhibiting ER activity.

*Functional assay vs. binding assay***.** Many steroid hormones, including testosterone, progesterone and their analogs, showed no affinity to ER in the binding assay but acted as agonists in the BG1 ER-luc assay or both reporter gene assays. Testosterone showed a 15 nM potency in the BG1 ER-luc assay and 10 µM in the HEK293 ER-bla assay. Androgens have been reported to activate the translocation of ER and induce the synthesis of the uterine-induced protein with barely detectable affinity to the cytosol ER[38,39]. Progesterone was identified as an active agonist in the BG1 ER-luc agonist mode assay with 4 µM potency, an active antagonist in the BG1 ER-luc antagonist mode assay with 58 µM potency, and was inactive in the HEK293 ER-bla assay. Progesterone has been reported to antagonize estrogen action not through interactions with ER but via progestin receptors[40,41]. Progesterone thus may require a full length receptor to exert its activity on ER. This would also explain its inactivity in the HEK293 ER-bla assay, which has only a partial receptor.

The retinoic acids (trans-retinoic acid and 13-cis retinoic acid) also showed no detectable affinity to ER in the binding assay, but acted as antagonists in the BG1 ER-luc assay and agonists in HEK293 ER-bla assay. Retinoic acid has been found previously to inhibit ER activity not through direct ER-binding but by altering the amount of ER protein bound to the ERE or affecting the transcriptional efficiency of this complex[27]. Another study in addition found that retinoic acids decreased ERα expression in estrogen-responsive endometrial cancer cells through crosstalk with the estrogen signaling pathway[28].

Table S1: qHTS assay protocol for HEK293 ER-Bla and BG1 ER-luc cells in agonist mode

		HEK293 ER-Bla		BG1 ER-luc	
Step	Parameter	Value	Description	Value	Description
1	Reagent	6 µL	5000 cells / well	5 µL	4000 cells / well

2	Incubation time	5 h	37°C, 5% CO_2	24 h	37°C, 5% CO_2
3	Compounds	23 nL	Dilution series	23 nL	Dilution series
4	Controls	23 nL	β-estradiol	23 nL	β-estradiol
5	Incubation time	18 h	37°C, 5% CO_2	22 h	37°C, 5% CO_2
6	Reagent	1 µL	CCF4 substrate	5 µL	ONE-Glo
7	Incubation time	2 h	Room Temperature	30 min	Room Temperature
8	Detection		Envision	20 sec	ViewLux

**Step**	**Notes**
1	1536-well black wall/clear bottom plates (Greiner Bio-One) for ERα-Bla cells and 1536-well white solid bottom plates (Greiner Bio-One) for BG1 Luc 4E2 cells using a Multidrop Combi (Thermo Fisher Scientific Inc.) with 8-tip dispense.
2	Pintool (Kalypsys) transfer of Tox21 compound library in columns of 5-48 for a (final) range of 1 nM to 92 µM (15 point titration) in the 5 µL assay volume and a range of 0.8 nM to 76 µM (15 point titration) in the 6 µL assay volume.
3	For ERα-Bla cell plates, pintool (Kalypsys) transfer of controls in columns 1-4. Column 1: three-fold sixteen-point β-estradiol titration starting at 3.83 µM to 0.1 pM; Column 2 and 3: β-estradiol of 40 nM and 20 nM respectively; Column 4: DMSO.<br>For BG1 Luc 4E2 cell plates, pintool (Kalypsys) transfer of controls in columns 1-4. Column 1: three-fold sixteen-point β-estradiol titration starting at 46 nM to 0.001 pM; Column 2: 10 nM β-estradiol; Column 3 and 4: DMSO.
4	Single tip dispense of detection reagent to each well using a Flying Reagent Dispenser (Aurora Discovery)

5 For ERα-Bla cell plates, fluorescence intensity at 460 and 530 nm emission was read at 405 nm excitation by an Envision (Perkin Elmer); for BG1 Luc 4E2 cell plates, luminescence intensity was read on a ViewLux (Perkin Elmer) at 20 sec exposure, gain high and 2X binning.

Table S2: qHTS assay protocol for ERα-Bla and BG1 Luc 4E2 cells in antagonist mode

		ERα-Bla		ER-BG1	
Step	Parameter	Value	Description	Value	Description
1	Reagent	5 µL	5000 cells / well	4 µL	4000 cells / well
2	Incubation time	5 h	37°C, 5% CO_2	24 h	37°C, 5% CO_2
3	Compounds	23 nL	Dilution series	23 nL	Dilution series
4	Controls	23 nL	4-hydroxy tamoxifen	23 nL	4-hydroxy tamoxifen
5	Reagent	1 µL	β-estradiol (stimulator)	1 µL	β-estradiol (stimulator)
6	Incubation time	18 h	37°C, 5% CO_2	21.5 h	37°C, 5% CO_2
7	Reagent	1 µL	CCF4 substrate	1 µL	CellTiter-Fluor
8	Incubation time	2 h	Room Temperature	30 min	37°C, 5% CO_2
9	Detection		Envision	2 sec	ViewLux
10	Reagent	4 µL	CellTiter-Glo	4 µL	ONE-Glo
11	Incubation time	0.5 h	Room Temperature	30 min	Room Temperature
12	Detection	1 sec	ViewLux	20 sec	ViewLux

Step Notes

1 1536-well black wall/clear bottom plates (Greiner Bio-One) for ERα-Bla cells and 1536-well white solid bottom plates (Greiner Bio-One) for BG1 Luc 4E2 cells using a Multidrop Combi (Thermo Fisher Scientific Inc.) with 8-tip dispense.

2 Pintool (Kalypsys) transfer of Tox21 compound library in columns of 5-48 for a (final) range of 1 nM to 92 µM (15 point titration) in the 5 µL assay volume and a range of 0.8 nM to 76 µM (15 point titration) in the 6 µL assay volume.

3 For ERα-Bla cell plates, pintool (Kalypsys) transfer of controls in columns 1-4. Column 1: two-fold sixteen-point 4-hydroxy tamoxifen titration starting at 1.0 µM to 0.01 nM; Column 2: top 16 wells with 1 µM of 4-hydroxy tamoxifen and bottom 16 wells with 76.6 µM of tetraoctyl ammonium bromide; Column 3 and 4: DMSO.

For BG1 Luc 4E2 cell plates, pintool (Kalypsys) transfer of controls in columns 1-4. Column 1: two-fold sixteen-point 4-hydroxy tamoxifen titration starting at 1.0 µM to 0.01 nM; Column 2: top 16 wells with 1 µM of 4-hydroxy tamoxifen and bottom 16 wells with 92 µM of tetra n-octyl ammonium bromide; Column 3 and 4: DMSO.

4 Single tip dispense of 0.5 nM (final) of β-estradiol (agonist/stimulator) by using a Flying Reagent Dispenser (Aurora Discovery) to each well except the bottom 16 wells of 3rd and 4th columns are dispensed with assay buffer.

5 Single tip dispense of detection reagent to each well using a Flying Reagent Dispenser (Aurora Discovery).

6 For ERα-Bla cell plates, fluorescence intensity at 460 and 530 nm emission was read at 405 nm excitation by Perkin Elmer's Envision; for BG1 Luc 4E2 cell plates, fluorescence intensity was read on Perkin Elmer's ViewLux.

7 Single tip dispense of detection reagent to each well using a Flying Reagent Dispenser (Aurora Discovery).

8 For ERα-Bla cell plates, luminescence intensity was read on Perkin Elmer's ViewLux at 1 sec exposure, gain high and 2X binning; for BG1 Luc 4E2 cell plates, luminescence intensity was read on Perkin Elmer's ViewLux at 20 sec exposure, gain high and 2X binning.

Table S3. Compound single channel activity outcome assignments based on curve rank and reproducibility

Curve rank	Reproducibility call	Activity outcome
>-1 and <1	inactive match	inactive
>-1 and <1	inconclusive	inconclusive
>=1	mismatch	inconclusive agonist
>=1	active match	active agonist
>4	inconclusive	active agonist
>=1 and <=4	inconclusive	inconclusive agonist
<=-1	mismatch	inconclusive antagonist
<=-1	active match	active antagonist
<-4	inconclusive	active antagonist
>=-4 and <=-1	inconclusive	inconclusive antagonist

Table S4. Compound assay activity outcome assignments based on multi channel readouts (a) HEK293 ER-bla agonist mode (b) HEK293 ER-bla antagonist mode (c) BG1 ER-luc antagonist mode

(a)

Ratio outcome	460 nm outcome	BLA 460 nm promiscuity	Auto fluorescence (blue) outcome	Activity outcome
inactive	N/A	N/A	N/A	inactive
inconclusive	N/A	N/A	N/A	inconclusive
active agonist	agonist	average curve rank ≤4	inactive or AC50 fluor/AC50 signal ≥3	active agonist

inconclusive agonist	agonist	average curve rank ≤4	inactive or AC50 fluor/AC50 signal ≥3	inconclusive agonist
agonist	agonist	average curve rank >4	agonist and AC50 fluor/AC50 signal <3	inconclusive agonist (fluorescent)
active antagonist	antagonist	N/A	N/A	active antagonist
inconclusive antagonist	antagonist	N/A	N/A	inconclusive antagonist

Abbreviations: AC50 fluor = AC50 in the auto fluorescence assay, AC50 signal = AC50 in the ratio channel of the ER-bla assay

(b)

Ratio outcome	460 nm outcome	Cell viability outcome	Other conditions	Activity outcome
inactive	N/A	N/A	N/A	inactive
inconclusive	N/A	N/A	N/A	inconclusive
active agonist	agonist	inactive or agonist	N/A	active agonist
active agonist	agonist	antagonist	AC50 viability/AC50 signal ≥3 ($p<0.05$)	active agonist
inconclusive agonist	agonist	N/A	N/A	inconclusive agonist
agonist	agonist	antagonist	AC50 viability/AC50 signal <3 or $p≥0.05$	inconclusive agonist (cytotoxic)
active antagonist	antagonist	inactive or agonist	N/A	active antagonist
active	antagonist	antagonist	AC50	active antagonist

antagonist			viability/AC50 signal ≥ 3 ($p<0.05$)	
inconclusive antagonist	antagonist	N/A	N/A	inconclusive antagonist
antagonist	antagonist	antagonist	AC50 viability/AC50 signal <3 or $p\geq 0.05$	inconclusive antagonist (cytotoxic)

Abbreviations: AC50 viability = AC50 in the cell viability assay, AC50 signal = AC50 in the ratio channel of the ER-bla assay

(c)

Antagonist mode outcome	Cell viability outcome	Other conditions	Activity outcome
inactive	N/A	N/A	inactive
inconclusive	N/A	N/A	inconclusive
active agonist	inactive or agonist	N/A	active agonist
active agonist	antagonist	AC50 viability/AC50 signal ≥ 3 ($p<0.05$)	active agonist
inconclusive agonist	N/A	N/A	inconclusive agonist
agonist	antagonist	AC50 viability/AC50 signal <3 or $p\geq 0.05$	inconclusive agonist (cytotoxic)
active antagonist	inactive or agonist	N/A	active antagonist
active antagonist	antagonist	AC50 viability/AC50 signal ≥ 3 ($p<0.05$)	active antagonist
inconclusive antagonist	N/A	N/A	inconclusive antagonist

antagonist	antagonist	AC50 viability/AC50 signal <3 or p≥0.05	inconclusive antagonist (cytotoxic)

Abbreviations: AC50 viability = AC50 in the cell viability assay, AC50 signal = AC50 in the BG1 ER-luc assay

Table S5. 39 ER reference chemicals and their activity outcomes in the ER qHTS assays

CAS	Name	Expected ER activity*	BG1 ER-luc agonist	HEK293 ER-bla agonist	BG1 ER-luc antagonist	HEK293 ER-bla antagonist
57-63-6	17alpha-Ethinylestradiol	Strong	active agonist	active agonist	inconclusive antagonist	inactive
56-53-1	Diethylstilbestrol	Strong	active agonist	active agonist	active antagonist	active antagonist
50-28-2	Estradiol	Strong	active agonist	active agonist	inconclusive antagonist	inconclusive
84-16-2	meso-Hexestrol	Moderate-Strong	active agonist	active agonist	active antagonist	inconclusive antagonist (cytotoxic)
57-91-0	17alpha-Estradiol	Moderate	active agonist	active agonist	active antagonist	inconclusive
131-55-5	2,2',4,4'-Tetrahydroxybenzophenone	Moderate	active agonist	active agonist	inactive	inconclusive agonist
53-16-7	Estrone	Moderate	inconclusive agonist	active agonist	inconclusive antagonist	inconclusive
68-22-4	Norethindrone	Moderate	active agonist	active agonist	inactive	inconclusive agonist

77-09-8	Phenolphthalein	Moderate	active agonist	inconclusive agonist	inconclusive agonist	active antagonist
68047-06-3	(Z)-4-Hydroxytamoxifen	Moderate Antagonist	active antagonist	inconclusive	active antagonist	active antagonist
68392-35-8	4-Hydroxytamoxifen	Moderate Antagonist	active antagonist	inconclusive agonist	active antagonist	active antagonist
50-41-9	Clomiphene citrate	Moderate Antagonist	active antagonist	inactive	active antagonist	active antagonist
129453-61-8	Fulvestrant	Moderate Antagonist	active antagonist	inactive	active antagonist	active antagonist
82640-04-8	Raloxifene hydrochloride	Moderate Antagonist	active antagonist	inconclusive agonist	active antagonist	active antagonist
10540-29-1	Tamoxifen	Moderate Antagonist	inconclusive antagonist	inconclusive agonist	active antagonist	active antagonist
58-18-4	17-Methyltestosterone	Weak	active agonist	active agonist	inconclusive antagonist	inactive
140-66-9	4-(1,1,3,3-Tetramethylbutyl)phenol	Weak	active agonist	active agonist	inconclusive antagonist	inconclusive antagonist (cytotoxic)
599-64-4	4-Cumylphenol	Weak	active agonist	active agonist	inconclusive antagonist	active antagonist

1987-50-4	4-Heptylphenol	Weak	active agonist	inactive	inactive	inactive
104-40-5	4-Nonylphenol	Weak	active agonist	inactive	inactive	inactive
84852-15-3	4-Nonylphenol, branched	Weak	active agonist	active agonist	inconclusive<br>antagonist	inconclusive
33228-44-3	4-Pentylaniline	Weak	inactive	inactive	active<br>agonist	inconclusive
80-05-7	Bisphenol A	Weak	active agonist	active agonist	inactive	active<br>antagonist
1478-61-1	Bisphenol AF	Weak	active agonist	active agonist	active<br>antagonist	inconclusive<br>antagonist<br>(cytotoxic)
77-40-7	Bisphenol B	Weak	active agonist	active agonist	inconclusive<br>antagonist	active<br>antagonist
94-26-8	Butylparaben	Weak	active agonist	active agonist	active<br>agonist	inconclusive
712-50-5	Cyclohexylphenylketone	Weak	inconclusive<br>agonist	inconclusive<br>agonist	inactive	inconclusive
446-72-0	Genistein	Weak	active agonist	active agonist	active<br>agonist	inconclusive<br>antagonist
143-50-0	Kepone	Weak	active agonist	active agonist	active<br>antagonist	inconclusive<br>antagonist

						(cytotoxic)
789-02-6	o,p'-DDT	Weak	active agonist	active agonist	inactive	inconclusive
56-55-3	Benz(a)anthracene	Negative-Weak Agonist (Metabolism Required)	active agonist	inconclusive agonist	inactive	active agonist
72-43-5	Methoxychlor	Negative-Weak Agonist (Metabolism Required)	active agonist	active agonist	inactive	active antagonist
486-66-8	Daidzein	Very Weak	active agonist	active agonist	active agonist	inconclusive agonist
120-47-8	Ethylparaben	Very Weak	active agonist	inconclusive agonist	inconclusive agonist	inconclusive agonist
50-22-6	Corticosterone	Negative	inactive	inactive	inactive	inactive
84-74-2	Dibutyl phthalate	Negative	inconclusive agonist	inconclusive agonist	inactive	inactive
57-83-0	Progesterone	Negative	active agonist	inactive	active antagonist	inconclusive
17980-47-1	Triethoxy(2-methylpropyl)silane	Negative	inactive	inactive	inactive	inactive

2943-75-1	Triethoxyoctylsilane	Negative	inconclusive agonist	inconclusive agonist	inactive	inactive

Abbreviations: DDT = dichlorodiphenyltrichloroethane

*These chemicals have been used to validate ER *in vitro* assays and were taken from the Organization for Economic Cooperation and Development (OECD) Test Guideline (TG) 457 BG1 guidance document[42]

Table S6. Tox21-88: 88 diverse compounds in the Tox21 library plated as duplicates in all screening plates that serve as internal controls for assay performance

CAS	Name
95-54-5	1,2-Phenylenediamine
65558-69-2	1,3-Diiminobenz(f)isoindoline
99-65-0	1,3-Dinitrobenzene
606-37-1	1,3-Dinitronaphthalene
102-06-7	1,3-Diphenylguanidine
2243-62-1	1,5-Naphthalenediamine
58-18-4	17-Methyltestosterone
86-87-3	1-Naphthaleneacetic acid
117-18-0	2,3,5,6-Tetrachloronitrobenzene
602-01-7	2,3-Dinitrotoluene
1421-63-2	2',4',5'-Trihydroxybutyrophenone
4460-86-0	2,4,5-Trimethoxybenzaldehyde
121-88-0	2-Amino-5-nitrophenol
6285-57-0	2-Amino-6-nitrobenzothiazole
2052-07-5	2-Bromobiphenyl
111-15-9	2-Ethoxyethyl acetate
149-30-4	2-Mercaptobenzothiazole
90-05-1	2-Methoxyphenol
534-52-1	2-Methyl-4,6-dinitrophenol
5307-14-2	2-Nitro-1,4-phenylenediamine
627-18-9	3-Bromo-1-propanol
140-66-9	4-(1,1,3,3-Tetramethylbutyl)phenol
94-82-6	4-(2,4-Dichlorophenoxy)butyric acid
80-07-9	4,4'-Dichlorodiphenyl sulfone
139-65-1	4,4'-Thiodianiline
119-34-6	4-Amino-2-nitrophenol
60-09-3	4-Aminoazobenzene
95-83-0	4-Chloro-1,2-diaminobenzene

106-47-8	4-Chloroaniline
74-11-3	4-Chlorobenzoic acid
100-01-6	4-Nitroaniline
1806-26-4	4-Octylphenol
98-29-3	4-tert-Butylcatechol
2835-95-2	5-Amino-2-methylphenol
148-24-3	8-Hydroxyquinoline
602-60-8	9-Nitroanthracene
584-79-2	Allethrin
446-86-6	Azathioprine
131860-33-8	Azoxystrobin
271-89-6	Benzofuran
80-05-7	Bisphenol A
188425-85-6	Boscalid
85-68-7	Butyl benzyl phthalate
56-75-7	Chloramphenicol
1861-32-1	Chlorthal-dimethyl
132-60-5	Cinchophen
120-32-1	Clorophene
117-81-7	Di(2-ethylhexyl) phthalate
77-73-6	Dicyclopentadiene
60-57-1	Dieldrin
111-77-3	Diethylene glycol monomethyl ether
744-45-6	Diphenyl isophthalate
97-77-8	Disulfiram
27176-87-0	Dodecylbenzenesulfonic acid
141-43-5	Ethanolamine
13194-48-4	Ethoprop
140-56-7	Fenaminosulf
22224-92-6	Fenamiphos
525-82-6	Flavone

66332-96-5	Flutolanil
10025-82-8	Indium trichloride
700-06-1	Indole-3-carbinol
121-75-5	Malathion
104206-82-8	Mesotrione
950-37-8	Methidathion
72-43-5	Methoxychlor
78415-72-2	Milrinone
4376-20-9	Mono(2-ethylhexyl) phthalate
110-26-9	N,N'-Methylenebisacrylamide
495-18-1	N-Hydroxybenzamide
59-87-0	Nitrofurazone
10552-74-6	Nitrothal-isopropyl
550-44-7	N-Methylphthalimide
19044-88-3	Oryzalin
527-20-8	Pentachloroaniline
1763-23-1	Perfluorooctane sulfonic acid
92-84-2	Phenothiazine
51-03-6	Piperonyl butoxide
29420-49-3	Potassium nonafluoro-1-butanesulfonate
94-13-3	Propylparaben
129-00-0	Pyrene
25103-58-6	tert-Dodecanethiol
52-24-4	Thiotepa
87820-88-0	Tralkoxydim
55219-65-3	Triadimenol
55335-06-3	Triclopyr
3380-34-5	Triclosan
131983-72-7	Triticonazole

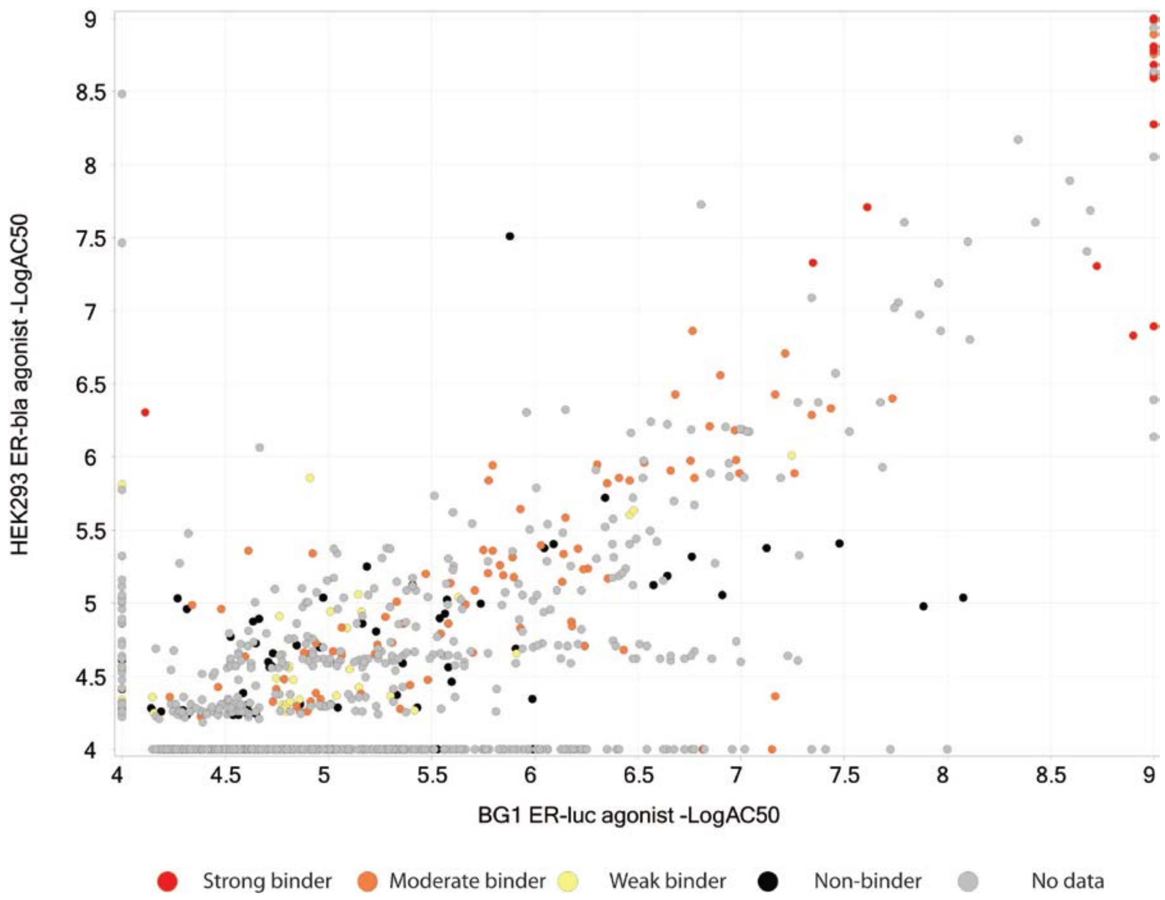

Supplementary Figure 1. –LogAC50 values of samples classified as active agonist in either the ER-BG1-luc or HEK293 ER-bla agonist mode assays. Data points are colored by activity in the ER binding assay[43] where strong binders (logRBA>0) are colored red, moderately strong binders (logRBA≥-3) are colored orange, weak binders (logRBA<-3) are colored yellow, non-binders are colored black, and compounds with no binding data are colored gray.

References

1 Huang, R. *et al.* Chemical genomics profiling of environmental chemical modulation of human nuclear receptors. *Environ Health Perspect* **119**, 1142-1148, doi:10.1289/ehp.1002952 (2011).

2 Hill, A. V. The possible effects of the aggregation of the molecules of haemoglobin on its dissociation curves. *J. Physiol. (London)* **40**, 4-7 (1910).

3 Inglese, J. *et al.* Quantitative high-throughput screening: a titration-based approach that efficiently identifies biological activities in large chemical libraries. *Proc Natl Acad Sci U S A* **103**, 11473-11478, doi:0604348103 [pii]
10.1073/pnas.0604348103 (2006).

4 PubChem. *PubChem BioAssay*, <http://www.ncbi.nlm.nih.gov/pcassay> (2013).

5 Kamiya, M., Toriba, A., Onoda, Y., Kizu, R. & Hayakawa, K. Evaluation of estrogenic activities of hydroxylated polycyclic aromatic hydrocarbons in cigarette smoke condensate. *Food Chem Toxicol* **43**, 1017-1027, doi:S0278-6915(05)00066-9 [pii]
10.1016/j.fct.2005.02.004 (2005).

6 St Helen, G. *et al.* Exposure and kinetics of polycyclic aromatic hydrocarbons (PAHs) in cigarette smokers. *Chem Res Toxicol* **25**, 952-964, doi:10.1021/tx300043k (2012).

7 Soares, A., Guieysse, B., Jefferson, B., Cartmell, E. & Lester, J. N. Nonylphenol in the environment: a critical review on occurrence, fate, toxicity and treatment in wastewaters. *Environ Int* **34**, 1033-1049, doi:S0160-4120(08)00008-1 [pii]
10.1016/j.envint.2008.01.004 (2008).

8 Routledge, E. J. & Sumpter, J. P. Structural features of alkylphenolic chemicals associated with estrogenic activity. *J Biol Chem* **272**, 3280-3288 (1997).

9 Safe, S. H. *et al.* Toxicology of environmental estrogens. *Reprod Fertil Dev* **13**, 307-315 (2001).

10 Tollefsen, K. E., Eikvar, S., Finne, E. F., Fogelberg, O. & Gregersen, I. K. Estrogenicity of alkylphenols and alkylated non-phenolics in a rainbow trout (Oncorhynchus mykiss) primary hepatocyte culture. *Ecotoxicology and Environmental Safety* **71**, 370-383 (2008).

11 Fertuck, K. C., Kumar, S., Sikka, H. C., Matthews, J. B. & Zacharewski, T. R. Interaction of PAH-related compounds with the alpha and beta isoforms of the estrogen receptor. *Toxicol Lett* **121**, 167-177, doi:S0378427401003447 [pii] (2001).

12 Fertuck, K. C., Matthews, J. B. & Zacharewski, T. R. Hydroxylated benzo[a]pyrene metabolites are responsible for in vitro estrogen receptor-mediated gene expression induced by benzo[a]pyrene, but do not elicit uterotrophic effects in vivo. *Toxicol Sci* **59**, 231-240 (2001).

13 Arcaro, K. F., O'Keefe, P. W., Yang, Y., Clayton, W. & Gierthy, J. F. Antiestrogenicity of environmental polycyclic aromatic hydrocarbons in human breast cancer cells. *Toxicology* **133**, 115-127 (1999).

14 Santodonato, J. Review of the estrogenic and antiestrogenic activity of polycyclic aromatic hydrocarbons: relationship to carcinogenicity. *Chemosphere* **34**, 835-848, doi:S0045-6535(97)00012-X [pii] (1997).
15 Hayakawa, K. *et al.* Estrogenic/antiestrogenic activities of quinoid polycyclic aromatic hydrocarbons. *Journal of Health Science* **57**, 274-280 (2011).
16 Liu, X., Ji, K. & Choi, K. Endocrine disruption potentials of organophosphate flame retardants and related mechanisms in H295R and MVLN cell lines and in zebrafish. *Aquat Toxicol* **114-115**, 173-181, doi:S0166-445X(12)00069-0 [pii]
10.1016/j.aquatox.2012.02.019 (2012).
17 Hashimoto, Y. & Nakamura, M. Estrogenic activity of dental materials and bisphenol-A related chemicals in vitro. *Dent Mater J* **19**, 245-262 (2000).
18 Cho, E. M. *et al.* Organotin compounds act as inhibitor of transcriptional activation with human estrogen receptor. *J Microbiol Biotechnol* **22**, 378-384, doi:JMB022-03-13 [pii] (2012).
19 Krause, M. *et al.* Sunscreens: are they beneficial for health? An overview of endocrine disrupting properties of UV-filters. *Int J Androl* **35**, 424-436, doi:10.1111/j.1365-2605.2012.01280.x (2012).
20 Morohoshi, K. *et al.* Estrogenic activity of 37 components of commercial sunscreen lotions evaluated by in vitro assays. *Toxicol In Vitro* **19**, 457-469, doi:S0887-2333(05)00011-1 [pii]
10.1016/j.tiv.2005.01.004 (2005).
21 Laws, S. C., Yavanhxay, S., Cooper, R. L. & Eldridge, J. C. Nature of the binding interaction for 50 structurally diverse chemicals with rat estrogen receptors. *Toxicol Sci* **94**, 46-56, doi:kfl092 [pii]
10.1093/toxsci/kfl092 (2006).
22 Salazar, K. D., Miller, M. R., Barnett, J. B. & Schafer, R. Evidence for a Novel Endocrine Disruptor: The Pesticide Propanil Requires the Ovaries and Steroid Synthesis to Enhance Humoral Immunity. *Toxicological Sciences* **93**, 62-74 (2006).
23 Yi, X. *et al.* Histone deacetylase inhibitor SAHA induces ERalpha degradation in breast cancer MCF-7 cells by CHIP-mediated ubiquitin pathway and inhibits survival signaling. *Biochem Pharmacol* **75**, 1697-1705, doi:S0006-2952(07)00732-0 [pii]
10.1016/j.bcp.2007.10.035 (2008).
24 De los Santos, M., Martinez-Iglesias, O. & Aranda, A. Anti-estrogenic actions of histone deacetylase inhibitors in MCF-7 breast cancer cells. *Endocr Relat Cancer* **14**, 1021-1028, doi:14/4/1021 [pii]
10.1677/ERC-07-0144 (2007).
25 Fiskus, W. *et al.* Hydroxamic acid analogue histone deacetylase inhibitors attenuate estrogen receptor-alpha levels and transcriptional activity: a result of hyperacetylation and inhibition of chaperone function of heat shock protein 90. *Clin Cancer Res* **13**, 4882-4890, doi:13/16/4882 [pii]
10.1158/1078-0432.CCR-06-3093 (2007).

26 Duong, V. *et al.* ERalpha and ERbeta expression and transcriptional activity are differentially regulated by HDAC inhibitors. *Oncogene* **25**, 1799-1806, doi:1209102 [pii]
10.1038/sj.onc.1209102 (2006).
27 Demirpence, E., Pons, M., Balaguer, P. & Gagne, D. Study of an antiestrogenic effect of retinoic acid in MCF-7 cells. *Biochemical and biophysical research communications* **183**, 100-106 (1992).
28 Li, X.-H., Li, H., Xiao, Z.-J. & Piao, Y.-S. Divergent effects of retinoic acids on the expression of ERÎ± and 17Î²-hydroxysteroid dehydrogenase type 2 in endometrial carcinoma cells (RL 95-2). *Journal of Clinical Endocrinology and Metabolism* **87**, 640-649 (2002).
29 Graupner, G. *et al.* 6-Substituted naphthalene-2-carboxylic acid analogs, a new class of retinoic acid receptor subtype-specific ligands. *Biochem. Biophys. Res. Commun.* **179**, 1554-1561 (1991).
30 Teng, M., Duong, T. T., Klein, E. S., Pino, M. E. & Chandraratna, R. A. Identification of a retinoic acid receptor alpha subtype specific agonist. *J Med Chem* **39**, 3035-3038 (1996).
31 Tobita, T. *et al.* Treatment with a new synthetic retinoid, Am80, of acute promyelocytic leukemia relapsed from complete remission induced by all-trans retinoic acid. *Blood* **90**, 967-973 (1997).
32 Okubo, T., Yokoyama, Y., Kano, K., Soya, Y. & Kano, I. Estimation of estrogenic and antiestrogenic activities of selected pesticides by MCF-7 cell proliferation assay. *Archives of Environmental Contamination and Toxicology* **46**, 445-453 (2004).
33 Klayman, D. L. Qinghaosu (artemisinin): an antimalarial drug from China. *Science* **228**, 1049-1055 (1985).
34 Sundar, S. N., Marconett, C. N., Doan, V. B., Willoughby, J. A., Sr. & Firestone, G. L. Artemisinin selectively decreases functional levels of estrogen receptor-alpha and ablates estrogen-induced proliferation in human breast cancer cells. *Carcinogenesis* **29**, 2252-2258, doi:bgn214 [pii]
10.1093/carcin/bgn214 (2008).
35 Horwitz, K. B. & McGuire, W. L. Actinomycin D prevents nuclear processing of estrogen receptor. *Journal of Biological Chemistry* **253**, 6319-6322 (1978).
36 Punchihewa, C., De Alba, A., Sidell, N. & Yang, D. XR5944: A potent inhibitor of estrogen receptors. *Molecular cancer therapeutics* **6**, 213-219 (2007).
37 Martinez-Campa, C. *et al.* Effect of Vinca alkaloids on ERÎ± levels and Estradiol-induced responses in MCF-7 cells. *Breast Cancer Research and Treatment* **98**, 81-89 (2006).
38 Schmidt, W. N., Sadler, M. A. & Katzenellenbogen, B. S. Androgen-uterine interaction: nuclear translocation of the estrogen receptor and induction of the synthesis of the uterine-induced protein (IP) by high concentrations of androgens in vitro but not in vivo. *Endocrinology* **98**, 702-716 (1976).
39 Rochefort, H. & Garcia, M. Androgen on the estrogen receptor. I - Binding and in vivo nuclear translocation. *Steroids* **28**, 549-560 (1976).

40 Koseki, Y., Zava, D. T., Chamness, G. C. & McGuire, W. L. Progesterone interaction with estrogen and antiestrogen in the rat uterus--receptor effects. *Steroids* **30**, 169-177 (1977).
41 Schwartz, S. M., Blaustein, J. D. & Wade, G. N. Inhibition of estrous behavior by progesterone in rats: role of neural estrogen and progestin receptors. *Endocrinology* **105**, 1078-1082 (1979).
42 OECD. *OECD Guidelines for the Testing of Chemicals* (OECD Publishing, 2012).
43 Tong, W. *et al.* Assessment of prediction confidence and domain extrapolation of two structure-activity relationship models for predicting estrogen receptor binding activity. *Environ Health Perspect* **112**, 1249-1254 (2004).
